# Injustices in Black Maternal Health: A Call for Different Research Questions, Orientations, and Methodologies

**DOI:** 10.3389/fpubh.2022.860850

**Published:** 2022-04-18

**Authors:** Shawnita Sealy-Jefferson

**Affiliations:** College of Public Health, Division of Epidemiology, The Ohio State University, Columbus, OH, United States

**Keywords:** Black women, maternal health, mass incarceration, violent death, epidemiology, community-based participatory action research

## Abstract

For decades, Black mothers have been most likely to suffer the worst outcomes of pregnancy, including death. Even though traditional individual level risk factors do not explain racial inequities in maternal morbidity, most studies identify Black race as a predictor, instead of the ways in which our society is structured around racism that makes Black mothers vulnerable to adverse health outcomes. As an example, the U.S is exceptional in incarcerating its residents, and Black men are six times and Black women are three times more likely than their white counterparts to be incarcerated. Relatedly, violent death caused by homicides disproportionately impacts Black communities, such that is the leading cause of death for males and females aged 10–34 years. Estimates suggest that more than 50% of urban residents know more than 10 murder victims, and approximately 200 people are affected by each neighborhood murder. Recent research has begun to shed light on the impacts of stressful neighborhood social conditions on risk of the adverse birth outcomes among Black mothers however, few studies have quantified the impact of macro-social neighborhood factors like violent death exposures and mass incarceration on Black maternal health. Future research that leverages relevant theoretical frameworks, is co-created and co-led with affected communities, and focuses on relevant neighborhood level traumas is warranted if we are to address the longstanding racial inequities in maternal health.

## Introduction

**“If you are silent about your pain, they'll kill you and say you enjoyed it.” -Zora Neale Hurston** **(****1****)**.

If maternal mortality is the tip of the iceberg then maternal morbidity is the base (2, 3). We can understand maternal health as a continuum, with optimal pregnancy outcomes for the mother on one end of the spectrum, and maternal mortality on the other (4, 5). Over the past several decades, maternal mortality has increased nearly two-fold (3), and the rising prevalence of chronic conditions (including obesity, hypertension, and diabetes) as well as cesarean births are likely contributing causes (6). For instance, pregnancy can worsen pre-existing conditions and increase risk for pregnancy complications such as preeclampsia, severe maternal morbidities like heart attack, and the worse outcome of pregnancy- maternal death (7). Pregnancy normally causes increased cardiac output, heart rate, and blood volume, all of which can cause cardiac strain (8). Cardiovascular disease is now the leading cause of maternal mortality (9), and mothers who endure and survive complications of pregnancy, like preeclampsia, have increased risk of long-term metabolic and cardiovascular disease (10–13).

## Racial Inequities

Black people are at least 3 times more likely to die from pregnancy related causes than white people, across all age groups (14). From epidemiologic studies we know that Black mothers are also more likely than white mothers to have maternal morbidities, irrespective of the varied definition of “morbidity” across studies (9). Black people are disproportionately burdened by cardiovascular disease risk factors and myocardial infarction during pregnancy, (15) as well as more severe peripartum cardiomyopathy disease (at diagnosis and unfortunately even 6 and 12 months post-diagnosis) (16). Data from the Nationwide Inpatient Sample suggests that Black women are also disproportionately burdened by cerebrovascular events in the peripartum period (17, 18), as well as severe pulmonary complications (19). From 1997 to 2014, severe maternal morbidity increased by 179% in Black women compared to 163% in white women (20). Further, Black women have higher rates of hospital readmission (21, 22), pregnancy associated hospitalization (23), and emergency department visits during the 90 days after delivery (24). Notably, studies have consistently identified racial inequities in maternal mortality across racial groups, after accounting for biomedical, sociodemographic, and behavioral factors (25, 26). The racial inequities in maternal health have been sufficiently documented in analyses comparing Black to other populations. Going forward, novel within-group analyses (comparing Black people to Black people) (27, 28) to identify policy and intervention relevant structural determinants of poor health (29) as well as intervening pathways and protective factors within the groups that have been made vulnerable to race, class, and gender oppression are warranted.

## The Social Context as an Overlooked Determinant

Social context, which can be understood as the social and political drivers of hierarchies and social stratification, including but not limited to policies and macroeconomic factors (30), has not been the focus of the majority of extant research on racial inequities in maternal health. Unjust exposure to health-harming macro-social factors are likely important drivers of the disproportionate burden of poor health in Black communities (31, 32). Research on determinants of poor maternal health across racialized groups overwhelmingly focuses on individual-level comorbidities (33). Few existing studies examine or acknowledge the relationship between racial inequities in maternal health and structural racism, which includes the social policies, institutional practices, cultural depictions, and other norms that reinforce, uphold, and perpetuate racial inequities (34). This is an important gap in the literature on this topic, especially given evidence that Black people have lower prevalence of five of the common high-risk pregnancy complications, yet have between 2.4–3.3 times higher likelihood of death due to these complications, compared to white people (19).

## Unjust Exposure to Mass Incarceration

Social determinants that are a function of racism and specifically and unequally burden Black people have not been examined as risk factors of poor maternal outcomes using within group analyses. For instance, exposures to “mass incarceration,” which refers to the extreme historical and contemporary levels of incarceration, occurrences that are so concentrated in communities of color that it becomes a common stage of in life-course (35). Approximately 50% of Black women have an imprisoned relative, compared to only 12% of their white counterparts (36). Further, Black people are more likely than the overall population to know an incarcerated individual, and to have a neighbor or an intimate partner incarcerated (36). Women make up 83% of those responsible for the costs associated with family member's court costs, which results in a financial burden that compounds any existing struggles to meet basic material needs (37). Direct and indirect contact with the criminal justice system exposes millions of Black women to health harming stressors that threaten their health and that of their families. Recent work suggests that women with experiences of incarceration are more likely to suffer premature mortality than those never incarcerated (38). Further, women (but not men) who have an incarcerated relative have been shown to have higher risk of obesity, heart attack, stroke, and fair or poor health, than those who do not (39). Despite specific calls for research on the life-course influences of mass incarceration on the health of Black people and communities (40), few studies have quantified the direct or contextual effect of mass incarceration on poor health and mortality within this group (41), and none have examined its effect on Black maternal health. This distinct over-exposure to incarceration that Black communities experience may be an important contributor to maternal health inequities and research and action to address this crisis is needed (36, 41).

## Unjust Exposure to Family and Community Violent Deaths

More than seven people suffer a violent death every hour, in the United States (42). Homicides disproportionately affect Black populations, such that they are the leading cause of death for Black males and females aged 10–34 (43, 44). Research using a community survey found that over half of urban respondents knew more than 10 murder victims, and approximately 200 people are affected by each neighborhood murder (45). While studies have examined the impact of neighborhood crime on adverse birth outcomes (45, 46), none have examined the unique contribution of neighborhood violent death exposures on Black maternal health. One study examined the relationship between neighborhood crime and hypertensive disorders of pregnancy using electronic health records linked to police-reported crime incidents, and found null results, likely due to exposure measurement error (47). Indeed, stress from losing a family or community member to violence may negatively impact health promoting behaviors, and poverty and racism likely exacerbate these associations (48). Community, (including state-sanctioned) violence is a public health issue that unjustly affects Black women, who are victims, witnesses, and grieving wives, girlfriends, and mothers of homicide victims. Even when Black people do not experience losing a close relative to violent death, which is rare in many urban areas, the hypervigilance caused by the constant threat of violence negatively impacts the quality of life, mental, and physical health of these people.

## Historical and Contemporary Redlining as a Root Cause of Toxic Stress

Residence in disadvantaged neighborhoods is a psychological and physiological stressor (49–51), because neighborhood exposures like social disorder, defined as “visible cues indicating a lack of order and social control” (51), are stress-inducing. Indeed, stressors originating from the neighborhood context are an important contributor to total stress load (52). The “broken windows” theory of urban decline suggests that public disorder causes urban decay and serious crime, and is predictive of poor mental and physical health (53). Black women are more likely to live in disadvantaged neighborhoods throughout their life-course (54), and to experience various family traumas (55). Our understanding of whether and how neighborhoods matter for health has been constrained because much of the literature uses sociodemographic variables from administrative data sources (like from U.S. Census), which may not equal the true neighborhood construct of interest (for example neighborhood disorder or community social ties) (56). Further, there is wide variability in the neighborhood measures used across studies, as is the level of aggregation (census tract, zip codes, block groups, etc.) which makes it difficult to identify what specific neighborhoods characteristics (and at what scale) should be the focus of interventions (56). Unfortunately, we have limited existing data on the predictive ability of structural racism, as manifested by community-level mass incarceration and the community trauma of violent deaths on Black maternal health (26). Nuanced and multi-level quantitative and qualitative (57) evidence on the associations between various manifestations of structural racism on Black maternal health will make it possible to target interventions and policy initiatives at critical periods of exposure across the life-course.

## Theory Can Help us Ask Different Research Questions and Find the Right Solutions

Reproductive Justice (RJ), conceptualized by Women of African Descent for Reproductive Justice in 1994, is a concept that can be understood as the merging together of reproductive rights and social justice (58). RJ is defined as the interconnected human rights to: (1) have children under the circumstances of one's choosing, (2) not have children, and (3) parent children in safe and healthy communities that are free from individual and state violence (58). Intersectionality, coined by Kimberle' Crenshaw, offers a framework for understanding the unique intersection of racial and gender oppressions experienced by Black women (59). RJ praxis elaborates how activism around bodily autonomy and intersectionality are connected, and facilitates status quo disruption. RJ articulates that the ability of people to determine their own reproductive destiny is directly influenced by the conditions of their community (60). RJ focuses on organizing women, girls, and their communities to resist structural power inequalities through a complete and transformative process of empowerment, one that improves lives of women, ensures healthy families, and sustainable communities (60). Ecosocial theory of disease distribution (61, 62), suggests that: (1) inequitable racial hierarchies prioritize groups who claim superiority at the expense of those deemed inferior; (2) race is reified as biology to establish racial categories; and (3) *inequitable living and working environments* facilitate the biological expression of racism and produce racial inequities in health through embodiment (61, 63–65). The ecosocial approach is guided by the question “who and what drives current and changing patterns of social inequalities in health” (62). The principal focus of this theory is how individuals biologically express exposures occurring from societal and ecological contexts. These frameworks have rarely been integrated to understand the linkages between community trauma and the disproportionate burden of adverse maternal health among Black people, but they can help us understand and most importantly intervene on these multi-level, macro-social determinants and move us toward maternal health equity.

## Discussion

Community-based participatory research (CBPR) projects are a critical approach for research on the social-structural determinants of health inequity, and are a crucial part of dismantling oppressive structures (66–68). CBPR projects that focus on associations between macro-social exposures including (but not limited to) community trauma caused by mass incarceration and violent deaths and maternal health of Black people (using within group analyses) are urgently needed. The COVID-19 pandemic, which highlighted and exacerbated longstanding racial inequities in health and resource distribution, should make it clear why research on racialized communities that is not grounded in relevant theories and does not center the lived experience and various ways of knowing of affected communities in the conception, design, implementation, and dissemination stages will cause more harm than good. Given this, research that is focused on the liberation of oppressed communities, and is led by members of affected communities (as equal thought leaders) should be prioritized for funding by local and national funders and philanthropic organizations. This perspective calls for different research questions- ones that are not bound by the current available data, are not based solely the intellectual curiosity of researchers, are informed by relevant theories and frameworks, and use participatory research methodologies *for action*.

## Data Availability Statement

The original contributions presented in the study are included in the article/supplementary material, further inquiries can be directed to the corresponding author.

## Author Contributions

SS-J conceived of, wrote, and edited the manuscript.

## Funding

Support for this manuscript was provided in part by the Robert Wood Johnson Foundation (Grant Number 77771). The views expressed here do not necessarily reflect the views of the Foundation.

## Conflict of Interest

The author declares that the research was conducted in the absence of any commercial or financial relationships that could be construed as a potential conflict of interest.

## Publisher's Note

All claims expressed in this article are solely those of the authors and do not necessarily represent those of their affiliated organizations, or those of the publisher, the editors and the reviewers. Any product that may be evaluated in this article, or claim that may be made by its manufacturer, is not guaranteed or endorsed by the publisher.
